# Determinants of dental treatment avoidance: findings from a nationally representative study

**DOI:** 10.1007/s40520-020-01652-7

**Published:** 2020-08-04

**Authors:** Kristin Spinler, Ghazal Aarabi, Carolin Walther, Richelle Valdez, Guido Heydecke, Elzbieta Buczak-Stec, Hans-Helmut König, André Hajek

**Affiliations:** 1 Department of Prosthetic Dentistry, Center for Dental and Oral Medicine, University Medical Center Hamburg-Eppendorf, Martinistraße 52, 20247 Hamburg, Germany; 2 Center Psychosocial Medicine, Institute of Medical Sociology, University Medical Center Hamburg-Eppendorf, Hamburg, Germany; 3 Department of Health Economics and Health Services Research, Hamburg Center for Health Economics, University Medical Center Hamburg-Eppendorf, Hamburg, Germany

**Keywords:** Dental avoidance, Dental visits, Health care utilization, Dental care, Dental health services, Determinants of oral health

## Abstract

**Background:**

Oral health care of older adults is of rising importance due to ongoing demographic changes. There is a lack of studies examining the determinants of dental treatment avoidance in this age group. Therefore, the objective of this study was to identify those determinants.

**Methods:**

Cross-sectional data were drawn from the second wave (year 2002) of the German Ageing Survey which is a population-based sample of community-dwelling individuals ≥ 40 years in Germany (*n* = 3398). Dental treatment avoidance was quantified using the question “Did you need dental treatments in the past twelve months, but did not go to the dentist?” [no; yes, once; yes, several times]. Socioeconomic and health-related determinants were adjusted for in the analysis. Multiple logistic regressions were performed.

**Results:**

In terms of need, 6.7% of individuals avoided dental treatment in the preceding twelve months. Multiple logistic regressions revealed that dental treatment avoidance was associated with younger age (total sample [OR 0.978; 95% CI 0.958–0.998] and men [OR 0.970; 95% CI 0.942–0.999]), unemployment (total sample [OR 1.544; 95% CI 1.035–2.302] and men [OR 2.004; 95% CI 1.085–3.702]), lower social strata (women [OR 0.814; 95% CI 0.678–0.977]), increased depressive symptoms (men [OR 1.031; 95% CI 1.001–1.062]), and increased physical illnesses (total sample [OR 1.091; 95% CI 1.006–1.183] and men [OR 1.165; 95% CI 1.048–1.295]). The outcome measure was not associated with income poverty, marital status and physical functioning.

**Conclusions:**

The present study highlights the association between dental treatment avoidance and different socioeconomic and health-related factors. These results suggest that it is necessary to promote the importance of dental visits.

## Background

The oral health care of older adults is of rising importance and high interest due to ongoing demographic changes that have resulted in a growing number of older adults with oral diseases [1]. It is predicted that there will be an increasing proportion and number of older adults, as well as an increased life expectancy, in Germany [2]. Positive changes in terms of the oral health status of older people have been seen, including an increase in the number of remaining teeth at end of life [1]. However, given the complexity of dental treatment required by the older population [3], dental research specific to this age group is required, in order to provide the best possible oral health care, as well as to avoid health inequities [1, 3].

Population-based dental research conducted as part of the German Oral Health Surveys (DMS) has observed poorer oral health and less frequent dental service utilization in older adults compared to younger age groups [4–6]. For instance, the fifth DMS showed an average DMFT-value of 11.2 (Mean number of Decayed, Missing, and Filled Permanent Teeth) in younger adults (35–44 years) and 17.7 in seniors (65–74 years) [1]. The need for routine dental visits to maintain and promote good oral health, in addition to personal oral hygiene and self-care, is well investigated and understood by oral health professionals [7]. Postponement or avoidance of dental visits, especially when treatment is needed, can have profound negative impacts on oral health, such as periodontitis and caries lesions, which can e.g. result in tooth loss [1]. Furthermore, poor oral health can negatively affect one’s physical health [6]. Clinical studies have shown that oral diseases are associated with systemic diseases due to a high inflammatory load, for instance, periodontal disease with atherosclerotic vascular disease [8–10]. Alongside these health consequences, poor oral health can negatively influence quality of life. For example, tooth loss can have impact on a persons’ chewing ability and facial appearance [11, 12].

A variety of factors that can influence regular dental care utilization have been elaborated in existing literature [7, 13, 14]. For instance, higher age was detected as a factor that negatively influences regular and preventive dental visits [7]. Further factors that could be found in younger and older adults are the presence of acute pain syndrome, education level, financial situation, gender, dental anxiety and fear, mental health status, the presence of functional limitations, among other things [7, 15, 16]. However, there are few studies that explore the factors that influence dental treatment avoidance among older adults. Dental treatment avoidance is a well-recognised problem in dental care [1, 4, 5]. Most existing studies evaluate the correlation between dental fear, oral health literacy or costs, and treatment avoidance in adults, but not in older adults specifically [16–20].

The results of the presented investigation might help to identify the influencing factors, with their help the oral self-care of older patients could be strengthened. Self-care is important for persons’ health in general. It is seen as being a helpful behavior of people that inter alia leads to a healthy life style, handles long-term conditions well and prevents illness or accidents [21, 22]. This is particularly important for older adults, whose health needs higher attention because of an aging body that is more susceptible to diseases [23]. The promotion of oral self-care for instance via educational and health promoting activities in this age group can lead to oral health-related [7, 24, 25] and furthermore to social and economic benefits [26].

## Aims

Therefore, the objective of this study was to identify determinants of dental treatment avoidance using data from a nationally representative sample of older adults. This is important because dental visits play an important role in promoting good oral health, and consequently general health.

## Methods

### Sample

Data from the second wave (year 2002) of the German Ageing Survey (DEAS) was used in this study. The Federal Ministry for Family Affairs, Senior Citizens, Women and Youth (BMFSFJ) funds the DEAS study. The DEAS study has a cohort-sequential design and is a representative study of non-institutionalized individuals ≥ 40 years. In the DEAS study, various topics are included, such as social support, health, or income.

4838 individuals from the birth cohorts 1911–1956 took part in the first wave in the year 1996 (50% response rate) and 5194 individuals took part in the second wave, which had a response rate of 38%. In the second wave, 1524 individuals were re-interviewed. Previous research has been shown that this response rate is similar to other large survey studies that have been conducted in Germany [27]. Refusal to participate and health reasons were main reasons for a lack of follow-up data. The DEAS study is ongoing. Further waves took place in 2008 (third wave), 2011 (fourth wave), 2014 (fifth wave) and 2017 (sixth wave). Further details about the DEAS study are provided elsewhere [28]. Because dental treatment avoidance was only quantified in the second wave of the survey, we only used data from the second wave of this study.

An ethical statement for the DEAS study was not required, as the criteria for it were not fulfilled (e.g., risk for the respondents, examination of patients or the use of invasive methods). Written informed consent was provided by all individuals.

### Dependent variable

The outcome measure was quantified using the question “Did you need dental treatments in the past twelve months but did not go to the dentist?” [no; yes, once; yes, several times]. It was dichotomized (0 = no; 1 = yes, once/yes, several times).

### Independent variables

Several independent variables were included in this study. With regard to socioeconomic variables, age, sex (stratified regressions), family status (married, and living together with spouse; married, and living separated from spouse; widowed; divorced; single), income poverty [60% median threshold (household net equivalent income)], occupational status (employed; retired; other: not employed), and social strata (according to [29]: 1 = lower class, 2 = lower middle class, 3 = middle class, 4 = upper middle class, 5 = upper class) were included. In addition, health-related variables were included, i.e. physical functioning (subscale physical functioning of the SF-36 [30]; from 0 = worst to 100 = best), depressive symptoms (15 item version of the Center for Epidemiological Studies Depression Scale [31], ranging from 0 to 45, in which higher values reflect more depressive symptoms), and the number of chronic illnesses, such as poor circulation or cancer (ranging from 0 to 11).

### Statistical analysis

Bivariate comparisons between (1) individuals who avoided dental treatment in the past 12 months and (2) individuals who did not avoid dental treatment in the past 12 months were made using independent *t* tests as well as Chi-squared tests, as appropriate. Subsequently, multiple logistic regressions were performed. The criterion for statistical significance was set at *p* < 0.05. Analyses were performed using Stata 15.1 (StataCorp, College Station, Texas, USA).

## Results

### Bivariate associations

In the analytic sample, 52.7% of the individuals were male and the average age was 59.6 years (± 11.4 years), ranging from 40 to 89 years. Amongst these, 6.7% of the individuals avoided dental treatment in the preceding twelve months, despite needing dental treatment. Bivariate associations between the explanatory variables and avoidance status are displayed in Table 1. Individuals who avoided dental treatment in the preceding 12 months had more depressive symptoms and more physical illnesses. Further details are displayed in Table 1.

**Table 1:** Sample characteristic stratified by dental treatment avoidance (no; yes, at least once in the previous 12 months) [*n* = 3398; wave 2 (year 2002)]

Variables	Individuals who did not avoid dental treatment in the preceding 12 months (in case of need) (*n* = 3172, 93.3%) *N* (%)	Individuals who avoided dental treatment in the preceding 12 months (in case of need) (*n* = 226, 6.7%) *N* (%)	Level of signi-ficance
Sex			
Male	1669 (93.2)	122 (6.8)	ns
Female	1503 (93.5)	104 (6.5)	
Family status			
Married, living together with spouse	2312 (93.3)	165 (6.7)	ns
Married, living separated from spouse	67 (94.4)	4 (5.6)	
Divorced	269 (93.1)	20 (6.9)	
Widowed	354 (94.2)	22 (5.8)	
Single	170 (91.9)	15 (8.1)	
Employment status			
Employed	1330 (93.9)	87 (6.1)	ns
Retired	1348 (93.6)	92 (6.4)	
Other: not employed	494 (91.3)	47 (8.7)	
Income poverty			
Yes	2874 (93.3)	205 (6.7)	ns
No	298 (93.4)	21 (6.6)	

	Mean (SD)	Mean (SD)	
Age in years	59.7 (11.5)	58.6 (10.5)	ns
Social strata	3.2 (1.1)	3.0 (1.1)	*
Depressive symptoms	7.4 (6.6)	8.7 (6.6)	**
Physical functioning	85.0 (22.3)	82.6 (23.1)	ns
Number of physical illnesses	2.2 (1.8)	2.5 (2.1)	*

Comparisons between the two groups were done using *t* test and chi-square procedures

*N* number, *SD* standard deviation, *ns* not significant

***p < 0.001, **p < 0.01, *p < 0.05, ^+^*p* < 0.10

### Regression analysis

The determinants of dental treatment avoidance are shown in Table 2. We tested for multicollinearity using the variance inflation criterion. We found that the largest variance was 1.54, indicating that we did not have a problem with multicollinearity.

**Table 2:** Determinants of dental treatment avoidance (0 = no; 1 = yes, at least once in the previous 12 months); results of multiple logistic regressions [wave 2 (year 2002)]

Independent variables	(1) Dental treatment avoidance—total sample	(2) Dental treatment avoidance—men	(3) Dental treatment avoidance—women
Age	0.978* (0.958–0.998)	0.970* (0.942–0.999)	0.986 (0.958–1.015)
Family status			
Married, living separated from spouse (Ref.: married, living together with spouse)	0.731 (0.260–2.050)	0.580 (0.134–2.521)	0.926 (0.214–4.004)
Divorced	1.018 (0.626–1.658)	1.147 (0.589–2.230)	0.880 (0.426–1.818)
Widowed	0.853 (0.517–1.408)	0.950 (0.407–2.219)	0.780 (0.408–1.490)
Single	1.108 (0.634–1.938)	1.217 (0.596–2.485)	0.898 (0.349–2.311)
Employment status			
Retired (reference: employed)	1.327 (0.825–2.133)	1.516 (0.791–2.906)	1.158 (0.573–2.340)
Other: not employed	1.544* (1.035–2.302)	2.004* (1.085–3.702)	1.338 (0.769–2.328)
Social strata (from 1 = lowest class to 5 = highest class)	0.914 (0.807–1.035)	1.018 (0.857–1.210)	0.814* (0.678–0.977)
Presence of income poverty (reference: absence of income poverty)	0.737 (0.447–1.215)	0.556 (0.259–1.195)	0.871 (0.445–1.703)
Depressive symptoms [from 0 (no depressive symptoms) to 45 (severe depressive symptoms)]	1.018^+^ (0.997–1.040)	1.031* (1.001–1.062)	1.007 (0.976–1.038)
Physical functioning [from 0 (worst) to 100 (best)]	0.998 (0.991–1.005)	1.001 (0.992–1.011)	0.995 (0.984–1.005)
Number of physical illnesses (from 0 to 11)	1.091* (1.006–1.183)	1.165** (1.048–1.295)	1.002 (0.881–1.140)
Constant	0.242* (0.0598–0.979)	0.163^+^ (0.0231–1.154)	0.421 (0.0550–3.232)
Observations	3.398	1.791	1.607
Pseudo *R*^2^	0.015	0.027	0.014

Odds ratios were reported; 95% confidence intervals in parentheses

***p < 0.001, **p < 0.01, *p < 0.05, ^+^*p* < 0.10

Multiple logistic regressions revealed that dental treatment avoidance was associated with younger age (total sample [OR 0.978; 95% CI 0.958–0.998] and men [OR 0.970; 95% CI 0.942–0.999]), unemployment (total sample [OR 1.544; 95% CI 1.035–2.302] and men [OR 2.004; 95% CI 1.085–3.702]), lower social strata (women [OR 0.814; 95% CI 0.678–0.977]), increased depressive symptoms (men [OR 1.031; 95% CI 1.001–1.062]), and increased physical illnesses (total sample [OR 1.091; 95% CI 1.006–1.183] and men [OR 1.165; 95% CI 1.048–1.295]). Dental treatment avoidance was not associated with income poverty, marital status and physical functioning.

## Discussion

### Main findings

The objective of this study was to identify determinants of dental treatment avoidance using a nationally representative sample of older adults in Germany. In this sample, 6.7% of the individuals avoided dental treatment in the preceding twelve months, despite needing dental treatment. Multiple logistic regressions showed that dental treatment avoidance was associated with younger age (total sample and men), unemployment (total sample and men), lower social strata (women), increased depressive symptoms (men), and increased physical illnesses (total sample and men). The outcome measure was not associated with income poverty, marital status and physical functioning.

### Previous research and possible explanations

Since the here presented data of the DEAS study was collected, no other German (and global) studies were published that focused on the determinants of dental treatment avoidance in particular. But in previous data, it has already been demonstrated that the frequency of dental visits decreases with increasing age [1, 4, 13]. The results of the presented study show that, within this age group of older adults (≥ 40 years), younger adults are more likely to avoid dental treatment than older adults, despite being aware of their treatment need. The decision not to visit a dentist, despite being aware of the need for treatment, is risky. Research to date presents heterogeneous results as to how the risk behaviour of a person changes over the lifecycle [32]. However, several studies suggest the willingness to take riskier decisions decreases with rising age [33–35]. We hypothesize that this could be a key reason why older participants are less likely to avoid dental treatment than younger participants.

The two trends, frequency of regular dental visits (previous research) on the one hand [1, 4, 13], and avoidance of needed treatment (present study) on the other, might run reversely with rising age. This observation is supported by results of another large population-based study, which detected that younger adults within the older age group (≥ 50 years) are more likely to postpone their dental visits due to financial reasons [20]. Although income poverty was not directly linked with our target variable, the important role of costs as a barrier to dental care utilization has been demonstrated in different surveys [36–38]. The presented results—at least partially—endorse this conclusion by detecting higher treatment avoidance among women in lower social strata, and those unemployed (in the total sample and men) as supporting factors for dental treatment avoidance. Both factors are associated with and willingness to pay extra health care costs [39–42].

One aspect that is mainly investigated when dental treatment avoidance was taken into account is dental anxiety or fear [16, 17]. This relation also might influence the present study results. Low social strata and unemployment are known to be associated with education level and health literacy [43–45]. Oral health literacy was previously observed to influence dental treatment avoidance. Namely, the higher oral health literacy, the lower treatment avoidance [18, 19]. This association might play a role in the present sample as well. Furthermore, it can be speculated that fear could play a role in this relationship. A low health literacy might facilitate fear and dental anxiety [46, 47], which previous studies have detected to be a strong influencing factor for dental treatment avoidance [16, 17]. The fact that the survey question included the awareness of treatment need (“Did you need dental treatments in the past twelve months but did not go to the dentist?”) may suggest that barriers like fear/anxiety lead people to not visit the dentist.

Male persons who had other diseases, such as depression or a number of physical illnesses (∅2.5), were also more likely to avoid dental treatment in the present survey. These patients may suffer from listlessness and lower self-care, which is associated with depression and the burden of different other diseases [48, 49]. Additionally, through psychological distress, there might be a shift of focus and personal time capacities towards these health problems and therefore away from oral health, as identified by a European survey by Holm-Petersen et al. [38].

There were potential gender differences detected in the present study, i.e. in some associated variables like depression, social strata, number of physical illness, as well as the utilization of dental services in general. Those differences were seen in other studies as well [1, 7, 13, 20]. However, the impact of gender on oral health is a less-studied phenomenon in dentistry to date. A study by Mc Grath and Bedi [50] found that women were more socially and psychologically affected by oral health problems than men. Additionally, women perceived that dental treatment could enhance their quality of life, general well-being, moods and appearance, while men did not. These two effects could explain why men are more likely than women to avoid dental treatment in our study.

### Strengths and limitations

This is the first study examining the determinants of dental treatment avoidance using a large nationally representative sample in Germany. The role of a wide range of determinants was investigated (e.g., socioeconomic and health-related factors). Only a small sample selection bias has been reported for the DEAS study [28]. Because this is a cross-sectional study, changes over time could not be examined. Dental treatment avoidance relied on self-reported data. Moreover, the exact reason for dental treatment avoidance (e.g., dental anxiety, little trust in the last visited dentist or financial reasons [51]) remains unknown and can therefore only be speculated upon. Thus, future research is required to provide a more detailed understanding.

## Conclusions

The present study highlighted the association between dental treatment avoidance and different socioeconomic and health-related factors. These results suggest that it is necessary to promote the importance of dental visits and communicate the risk of dental treatment avoidance, especially on channels that are accessible for middle-aged and older adults. There may be a need for campaigns that are gender sensitive and address older men and their oral health needs in particular. This study contributes to discussions around inequality, and the reduction of oral health inequality in Germany. There is a need for future studies that deliver more up-to-date data about the determinants of dental treatment avoidance.

## Availability of data and materials

The anonymized dataset for the second wave of the DEAS (2002) was retrieved from the Research Data Centre of the German Centre of Gerontology (https://www.dza.de/en/fdz/german-ageing-survey/access-to-deas-data.html), which is available to scientists at universities and research institutes for secondary analyses I.
